# Serum Levels of Surfactant Proteins in Patients with Combined Pulmonary Fibrosis and Emphysema (CPFE)

**DOI:** 10.1371/journal.pone.0157789

**Published:** 2016-06-23

**Authors:** Andriana I. Papaioannou, Konstantinos Kostikas, Effrosyni D. Manali, Georgia Papadaki, Aneza Roussou, Aris Spathis, Argyro Mazioti, Ioannis Tomos, Ilias Papanikolaou, Stelios Loukides, Kyriakos Chainis, Petros Karakitsos, Matthias Griese, Spyros Papiris

**Affiliations:** 1 2nd Respiratory Medicine Department, “Attikon” University Hospital, Athens Medical School, National and Kapodistrian University of Athens, Athens, Greece; 2 Department of Cytopathology, “Attikon” University Hospital, Athens Medical School, National and Kapodistrian University of Athens, Athens, Greece; 3 Department of Radiology, “Attikon” University Hospital, Athens Medical School, National and Kapodistrian University of Athens, Athens, Greece; 4 Respiratory Medicine Department, “Corfu General Hospital”, Corfu, Greece; 5 Hauner Children’s University Hospital, Ludwig-Maximilians-University, German Center for Lung Research, Lindwurmstrasse 4, 80337, Munich, Germany; Augusta University, UNITED STATES

## Abstract

**Introduction:**

Emphysema and idiopathic pulmonary fibrosis (IPF) present either per se or coexist in combined pulmonary fibrosis and emphysema (CPFE). Serum surfactant proteins (SPs) A, B, C and D levels may reflect lung damage. We evaluated serum SP levels in healthy controls, emphysema, IPF, and CPFE patients and their associations to disease severity and survival.

**Methods:**

122 consecutive patients (31 emphysema, 62 IPF, and 29 CPFE) and 25 healthy controls underwent PFTs, ABG-measurements, 6MWT and chest HRCT. Serum levels of SPs were measured. Patients were followed-up for 1-year.

**Results:**

SP-A and SP-D levels differed between groups (p = 0.006 and p<0.001 respectively). In post-hoc analysis, SP-A levels differed only between controls and CPFE (p<0.05) and CPFE and emphysema (p<0.05). SP-D differed between controls and IPF or CPFE (p<0.001 for both comparisons). In IPF SP-B correlated to pulmonary function while SP-A, correlated to the Composite Physiological Index (CPI). Controls current smokers had higher SP-A and SP-D levels compared to non-smokers (p = 0.026 and p = 0.023 respectively). SP-D levels were higher in CPFE patients with extended emphysema (p = 0.042). In patients with IPF, SP-B levels at the upper quartile of its range (≥26 ng/mL) presented a weak association with reduced survival (p = 0.05).

**Conclusion:**

In conclusion, serum SP-A and SP-D levels were higher where fibrosis exists or coexists and related to disease severity, suggesting that serum SPs relate to alveolar damage in fibrotic lungs and may reflect either local overproduction or overleakage. The weak association between high levels of SP-B and survival needs further validation in clinical trials.

## Introduction

Pulmonary emphysema and idiopathic pulmonary fibrosis (IPF) are two distinct entities defined by different clinical, functional, radiological, and pathological criteria [1]. IPF is the most common of the idiopathic interstitial lung diseases (i-ILDs) and has the histopathologic and/or radiologic pattern of usual interstitial pneumonia (UIP) [2], while emphysema is defined as an enlargement of the air spaces distal to the terminal bronchioles due to the destruction of tissues forming their walls [3]. These two entities coexist in a condition characterized by upper lobe emphysema and lower lobe pulmonary fibrosis, which is known as combined pulmonary fibrosis and emphysema (CPFE) [1, 4]. The coexistence of the two conditions, which have different pathophysiological and functional characteristics, results in the development of a disease entity, with distinct clinical and functional characteristics and different prognosis compared to its individual components [5].

Pulmonary surfactant is a highly surface-active mixture of proteins and lipids that is synthesized and secreted onto the alveoli by type II epithelial cells [6, 7]. The protein part of surfactant constitutes of four types of surfactant proteins (SP), SP-A, SP-B, SP-C and SP-D. SP-A and SP-D are hydrophilic proteins that regulate surfactant metabolism and have immunologic functions, whereas SP-B and SP-C are hydrophobic molecules, which play a direct role in the organization of the surfactant structure in the interphase and in the stabilization of the lipid layers during the respiratory cycle [8, 9]. Different polymorphisms of SP-A [10], and SP-B [10–13] genes and mutations in SP-C [14] genes have been related to COPD. Furthermore, studies have shown that circulating SP-A and SP-D levels were increased in patients with COPD compared to normal smokers and non-smoking controls and correlated to airway obstruction [15–17]. On the other hand, there is evidence showing a possible role of surfactant proteins in the development of ILDs. Infants and children with mutations in the genes encoding surfactant proteins develop such a disease early in their life [18], while mutations of SP-A and C genes are associated with familial interstitial lung disease [19–23]. Serum levels of SP-D have been shown to be higher in patients with IPF compared to control subjects [24, 25]. Furthermore, serum SP-D concentrations are related to the annual rate of deterioration of pulmonary function [26], while high serum levels of SP-A and SP-D seem to be predictors of rapid disease deterioration and have been associated with poor survival in patients with IPF [25, 27, 28, 29]. Finally, there is evidence than SP-C gene mutations are associated with CPFE [30–33], while SP-D has been reported to correlate to pulmonary function in these patients [34].

According to the above, we hypothesized that in patients with CPFE, SP would be more disturbed in comparison to patients with only IPF and patients with only emphysema in a clinically significant way. The aim of the present study was to evaluate serum levels of Surfactant Proteins (A, B, C and D) in patients with CPFE, emphysema only, IPF only, and healthy controls and to test their possible associations to pulmonary function, disease severity, and survival.

## Methods

### Subjects

From February 2013 to December 2014 we enrolled 147 consecutive patients (31 with emphysema, 62 with IPF, and 29 with CPFE) and 25 healthy controls (smokers and non-smokers). All study subjects were in stable condition and did not report any exacerbation or respiratory tract infection during the last 8 weeks. Patients with α_1_-antitrypsin deficiency, connective tissue disease at the time of diagnosis of CPFE, diagnosis of other interstitial lung disease (such as drug induced ILDs, pneumoconiosis, hypersensitivity pneumonitis, sarcoidosis, pulmonary histiocytosis, LAM, and eosinophilic pneumonia), patients unable to perform PFTs, as well as patients with inability or unwillingness to collaborate with the investigators were not included in the study. All subjects were followed up for one year and their vital status has been recorded.

### Study design

All patients provided a detailed history upon arrival, and underwent physical examination and blood samples collection. Subsequently they were submitted to pulmonary function tests, measurement of body mass index (BMI) and evaluation of exercise capacity using the 6 minutes walking test. Arterial blood gases were also measured. Finally, all patients underwent high resolution computed tomography of the chest (HRCT). The study protocol was approved by the ethics committee of “Attikon” University Hospital, Chaidari Athens Greece and all patients gave written informed consent.

### Pulmonary Function Tests

Pulmonary function tests (PFTs) were performed with commercially available system (Master Screen, Erich Jaeger GmbH, Wuerzburg, Germany) and included post-bronchodilator forced expiratory volume in one second (FEV_1_), FVC, FEV_1_/FVC ratio, total lung capacity (TLC), residual volume (RV), inspiratory capacity (IC) and diffusing capacity for carbon monoxide (DL_CO_). Diffusing capacity for carbon monoxide (DL_CO_) and diffusing capacity for carbon monoxide adjusted for alveolar volume (DL_CO_/V_A_) were assessed by means of the single breath method with the patient in the sitting position. Lung function measurements were expressed as percentages of predicted values. Tests were performed according to the American Thoracic Society guidelines [35] by the same technician in order to ensure consistency of the results.

The composite physiologic index that is associated to mortality in IPF and CPFE [36] was [calculated according to the following formula: 91.0—(0.65 x percent predicted diffusing capacity for carbon monoxide [DLCO])—(0.53 x percent predicted FVC) + (0.34 x percent predicted FEV_1_)][37] was determined in all subjects [38]. In all patients, arterial blood samples were taken for the measurement of PaO_2_ and PaCO_2_ using a commercially available blood gas analyzer (model 1630; Instrumentation Laboratories, Milan Italy)

### HRCT

All patients underwent HRCT using Brilliance CT 64-channel scanner (Philips, Eindhoven, the Netherlands). Scans were performed with 1–1.5mm section thickness and a 1–2 sec scanning time during breath holding at end inspiration. Films were read by a radiologist with expertise in HRCT who was blinded to the rest of the measurements. The degree of emphysema and/or fibrosis was calculated using a visual score as previously described [39, 40], using a five-point scale based on the percentage of lung involved: 0: no emphysema/fibrosis; 1: up to 25% of the lung parenchyma involved; 2: between 26–50% of lung parenchyma involved; 3: between 26–75% of the lung parenchyma involved; and 4 between 76–100% of lung parenchyma involved. Grades of the axial images of each lung were added and divided by the number of images evaluated to yield emphysema and/or fibrosis scores that ranged from 0 to 4 [39, 40]. The percentage of destructed lung area (%DLA) was obtained by summing %emphysema and %fibrosis [41]. The presence of emphysematous lesions ≥15% (i.e. score ≥0.6) of the pulmonary parenchyma in CPFE patients was considered as significant [42, 43]

### Assessment of Dyspnea and Exercise Capacity

Dyspnea was assessed with the modified (5-point) Medical Research Council (MRC) dyspnea scale, as previously described [44]. Exercise capacity was measured with the 6 minutes walk test in a 100 ft corridor, according to the ATS guidelines [45]. The distance walked in 6 minutes, difference in dyspnea according to Borg scale and alterations in oxygen saturation were recorded in each patient.

### Blood Samples Collection and Measurements of Surfactant Proteins

Blood samples were collected from each subject and were immediately centrifuged at 1500g for 15 min at 4°C and the supernatant (serum) was stored at -80°C until measurements.

Serum surfactant proteins were measured using commercially available enzyme-immunosorbent assay (ELISA) kits (SP-A, SP-B and SP- C, USCN Life Science, China and, SP-D, Bioventor, Germany) according to the manufacturer's protocol. The lower limits of detection were the following: SP-A: 8.2 pg/mL, SP-B: 0.31 ng/ml, SP-C: 0.112 ng/mL and SP-D: 0.01 ng/mL.

### Statistical analysis

Normality of distributions was checked with Kolmogorov-Smirnov test. Comparisons between groups were performed with Kruskal-Wallis tests, with appropriate post-hoc tests (Dunn's test). Correlations were assessed using Spearman’s rank correlation coefficient. For the avoidance of type I errors during the performance of multiple correlations we have used the correction method described by Benjamini and Hochberg (1995)[46] according to which a p-value<0.023 was considered statistically significant. Group data are expressed as mean±SD or as median (interquartile ranges) for normally distributed and skewed data, respectively. Kaplan Meier Curves were created in order to compare survival in patients with low and those with elevated SP levels and the groups were compared using Log Rank test. The 75^th^ percentile of each SP was used as a cut-off point in order to divide the patients in those with elevated SP levels and those with non-elevated SP levels. Graphical presentations of times to events were performed with Kaplan-Meier curves. Results are presented as hazard ratios (HR) with 95% confidence intervals (CI). With the exception of the multiple correlations, all other p-values <0.05 were considered statistically significant. Analysis was performed using SPSS 17 statistical package (SPSS, Chicago, IL) and GraphPad Prism 5 (GraphPad Software Inc, La Jolla, CA, USA).

## Results

**Fig 1:**
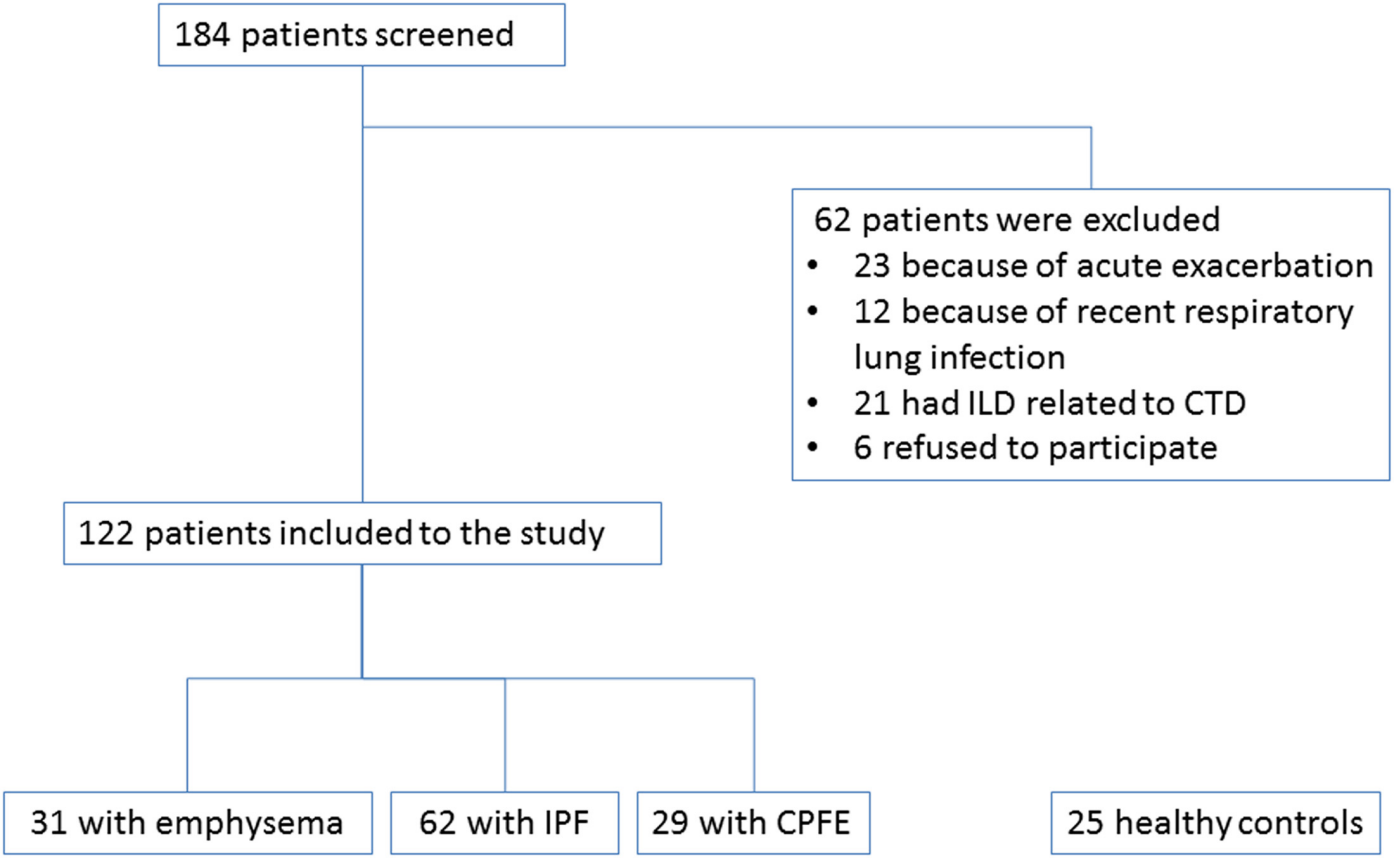
Flow chart showing subjects who were finally included to the study.

**Fig 2:**
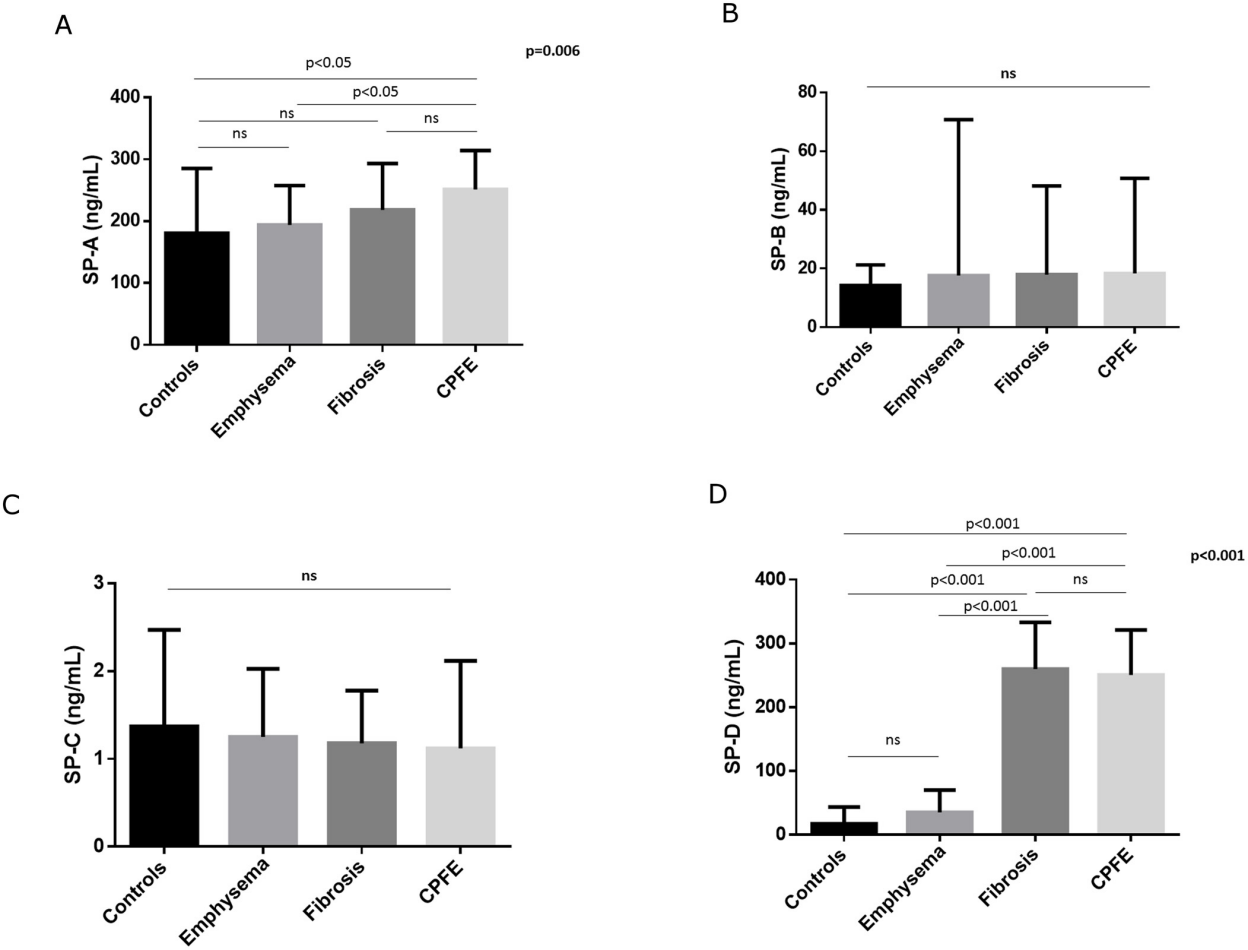
Serum surfactant protein levels in the different study groups. A. SP-A, B. SP-B, C. SP-C, D. SP-D.

We evaluated 184 consecutive patients with either emphysema, IPF and CPFE and 122 of them were eligible to be included in the present study. The flow chart of the study participants is shown in Fig 1. An additional 25 healthy subjects (smokers and non-smokers) were included as healthy controls. The demographic and functional characteristics as well as serum levels of surfactant proteins of the 122 patients and 25 healthy controls included in the study are presented in Table 1. Serum SP-A and SP-D levels significantly differed between groups (p = 0.006 and p<0.001 for SP-A and SP-D, respectively; Fig 2A–2D). In post-hoc analysis, SP-A levels differed significantly only between controls and CPFE (p<0.05) and between CPFE and emphysema (p<0.05). Similarly, post hoc analysis regarding serum SP-D levels revealed significant differences between controls and fibrosis or CPFE as well as emphysema and fibrosis or CPFE (p<0.001 for all comparisons).

**Table 1:** Demographic and functional characteristics of the study participants.

	Controls N = 25	Emphysema only N = 31	IPF only N = 62	CPFE N = 29	p value
**Age (years)**	**64.0(55.5, 67.5)**	**66.0 (59.0, 71.0)**	**72.0 (66.8, 78.0)**	**75.0 (63.0, 77.0)**	**<0.001**
**Gender (Female) N(%)**	**14 (56.0%)**	**7 (22.6%)**	**19 (30.6%)**	**3 (10.3%)**	**0.002**
**BMI kg/m**^**2**^	**26.7 (24.5, 29.5)**	**24.9 (21.5, 26.8)**	**27.2 (24.5, 29.6)**	**27.9 (23.6, 29.1)**	**0.005**
**Smoking (current/ex/never)**	**9/5/11**	**18/13/0**	**5/30/27**	**5/22/2**	**<0.001**
**Pys**	**10.0 (0.0, 35.0)**	**70.0 (40.0, 95.0)**	**12.5 (0.0, 36.0)**	**50.0 (30.0, 75.0)**	**<0.001**
**FEV**_**1**_ **(%pred)**	**92.0 (87.5, 99.0)**	**56.4 (33.7, 79.6)**	**76.4 (65.5, 88.1)**	**76.3 (69.5, 98.0)**	**<0.001**
**FVC (%pred)**	**90.0 (82.0, 93.5)**	**72.8 (60.1, 93.7)**	**68.3 (57.9, 82.1)**	**68.5 (60.3, 94.1)**	**0.001**
**FEV**_**1**_**/FVC**	**82.0 (78.5, 85.2)**	**54.4 (40.4, 69.0)**	**85.7 (81.3, 90.8)**	**79.0 (74.4, 86.7)**	**<0.001**
**DLCO (%pred)**	**85.0 (82.0, 88.0)**	**52.4 (36.5, 72.0)**	**46.8 (35.2, 62.9)**	**32.0 (22.8, 46.4)**	**<0.001**
**TLC (%pred)**	**85.0 (82.0, 87.5)**	**93.0 (81.2, 105.8)**	**59.1 (50.2, 73.2)**	**63.4 (56.5, 78.5)**	**<0.001**
**RV (%pred)**	**83.0 (80.5, 88.0)**	**119.0 (90.3, 126.5)**	**54.9 (43.1, 67.4)**	**57.4 (47.9, 80.5)**	**<0.001**
**FRC (%pred)**	**83.0 (81.0, 88.5)**	**100.0 (83.1, 129.8)**	**60.7 (52.9, 77.1)**	**67.0 (53.5, 80.0)**	**<0.001**
**PO**_**2**_**/FiO**_**2**_	**390.5 (380.9, 392.9)**	**333.3 (300.0, 371.4)**	**352.4 (321.4, 3711.4)**	**285.7 (258.6, 350.0)**	**<0.001**
**6MWD (m)**	**550.0 (497.5, 551.0)**	**455.0 (300.0, 515.0)**	**459.5 (330.0, 500.8)**	**207.0 (100.0, 424.5)**	**<0.001**
**DLA (%)**	**N/A**	**1.68 (0.6, 2.9)**	**1.5 (1.14, 2.2)**	**2.4 (1.75, 3.3)**	**0.001**
**CPI**	**20.9 (17.7, 24.9)**	**36.5 (21.4, 51.4)**	**49.4 (36.6, 60.2)**	**57.2 (48.3, 63.4)**	**<0.001**
**SP-A (ng/ml)**	**180.4 (118.9, 285.2)**	**193.9 (105.8, 257.6)**	**218.0 (148.5, 293.2)**	**251.2 (225.9, 314.1)**	**0.006**
**SP-B (ng/ml)**	14.3 (6.6, 21.3)	17.6 (7.2, 26.3)	17.9 (10.7, 25.9)	18.4 (11.9, 27.3)	0.353
**SP-C (ng/ml)**	1.4 (0.8, 2.2)	1.3 (0.7, 2.0)	1.2 (0.9, 1.8)	1.1 (0.8, 2.1)	0.925
**SP-D (ng/ml)**	**17.0 (7.1, 43.6)**	**35.2 (19.1, 70.1)**	**259.8 (196.7, 333.2)**	**250.7 (181.8, 321.0)**	**<0.001**

Abbreviations: BMI: Body Mass Index, FEV_1_: Forced expiratory Volume in one Second, FVC: Forced Exhaled Vital Capacity, DLCO: Diffusing Capacity for Carbon Monoxide, TLC: Total Lung Capacity, FRC: Functional Residual Capacity, RV: Residual Volume, 6MWD: 6 Minute Walking Distance, DLA: Destructed Lung Area, CPI: Composite Physiological Index, SP: Surfactant Protein, IPF: Idiopathic Pulmonary Fibrosis, CPFE: Combined Pulmonary Fibrosis and Emphysema. Bold represent statistically significant differences.

### Correlations of surfactant proteins and pulmonary function tests

**Fig 3:**
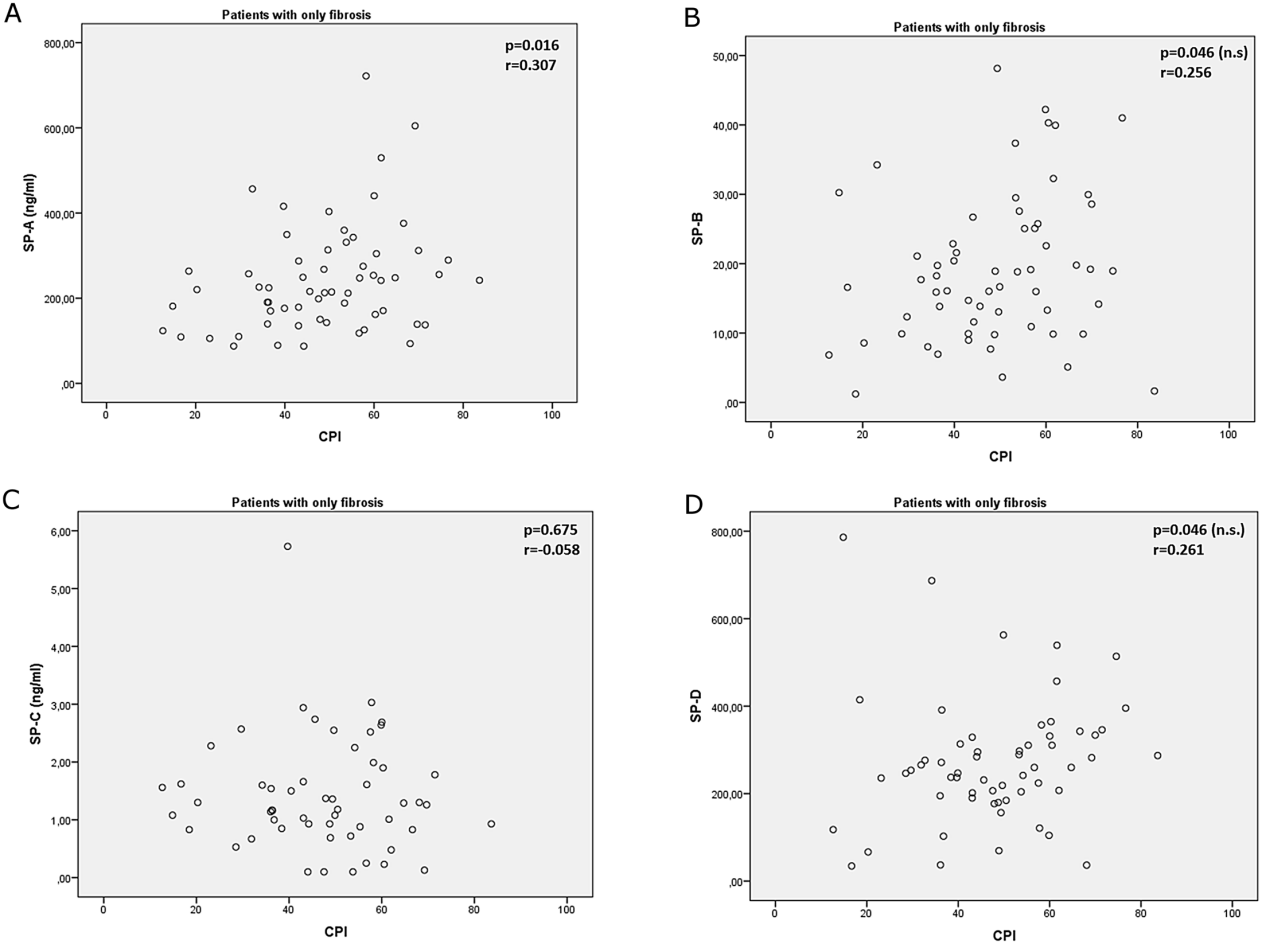
Composite Physiological Index (CPI) correlations to A. SP-A, B. SP-B, C. SP-C and D: SP-D. Level of statistical significance after correction for multiple correlation analysis was set at p < 0.023.

Correlations of surfactant proteins and pulmonary function are shown in Table 2. In patients with fibrosis serum SP-B levels correlated significantly to pulmonary function test results, and specifically to FEV_1_, FVC, DLCO, TLC, and FRC. The CPI presented only a weak correlation with SP-A (p = 0.016; Fig 3A–3D).

**Table 2:** 

	FEV_1_	FVC	FEV_1_/FVC	DLCO	TLC	RV	FRC	HRCT score	6MWD	PO_2_/FIO_2_	CPI
**SP-A**											
**Emphysema only**											
** p**	0.838	0.992	0.374	0.322	0.616	0.354	0.747	0.155	0.048	0.426	0.428
** r**	-0.039	-0.002	-0.168	-0.227	-0.099	-0.182	-0.065	0.271	-0.363	-0.156	0.150
**IPF**											
** p**	0.258	0.119	0.487	**0.025**	0.162	0.167	0.229	0.967	0.225	0.634	**0.016**
** r**	-0.147	-0.202	0.091	**-0.290**	-0.183	-0.181	-0.158	-0.005	-0.165	-0.062	**0.307**
**CPFE**											
** p**	0.338	0.385	0.812	0.215	0.466	0.105	0.071	0.575	0.063	0.349	0.136
** r**	-0.209	-0.190	0.052	-0.269	-0.160	-0.347	-0.383	-0.115	-0.393	-0.188	0.320
**SP-B**											
**Emphysema only**											
** p**	0.082	0.232	0.385	0.483	0.127	0.152	0.346	0.317	0.845	0.269	0.553
** r**	0.322	0.225	0.164	0.138	0.295	0.278	0.189	-0.189	0.037	0.205	-0.113
**IPF**											
** p**	**0.005**	**0.012**	0.429	**0.012**	**0.003**	0.042	**0.020**	0.798	0.192	0.030	0.046
** r**	**-0.358**	**-0.318**	-0.103	**-0.322**	**-0.378**	-0.263	**-0.300**	0.034	-0.177	-0.276	0.256
**CPFE**											
** p**	**0.006**	**0.003**	**0.010**	0.371	0.248	0.587	0.715	0.447	0.814	0.188	0.057
** r**	**0.538**	**0.570**	**-0.507**	0.187	0.240	-0.114	-0.077	-0.150	-0.050	0.252	-0.385
**SP-C**											
**Emphysema only**											
** p**	0.536	0.580	0.735	0.707	0.078	0.767	0.117	0.718	0.558	0.689	0.693
** r**	0.127	0.114	0.070	-0.079	0.359	0.062	0.321	0.074	-0.118	-0.081	-0.081
**IPF**											
** p**	0.610	0.337	0.047	0.963	0.298	0.685	0.850	0.847	0.987	0.752	0.675
** r**	0.071	0.133	0.271	-0.007	0.146	0.057	-0.027	-0.074	-0.002	0.044	-0.058
**CPFE**											
** p**	0.161	**0.009**	0.376	0.668	0.684	0.458	0.834	0.572	0.328	0.393	0.567
** r**	0.326	**0.571**	-0.209	-0.102	-0.097	-0.176	-0.050	0.124	0.231	0.187	-0.136
**SP-D**											
**Emphysema only**											
** p**	0.260	0.981	0.051	0.097	0.099	0.071	0.044	0.306	0.245	0.966	0.107
** r**	0.212	-0.005	0.360	-0.320	-0.318	-0.346	-0.391	0.193	-0.215	0.008	0.300
**IPF**											
** p**	0.212	0.072	0.088	0.106	0.033	**0.023**	0.047	0.240	0.908	0.910	0.046
** r**	-0.165	-0.236	0.224	-0.214	-0.281	**-0.299**	-0.262	0.158	-0.016	-0.015	0.261
**CPFE**											
** p**	0.784	0.575	0.508	0.444	0.243	0.798	0.352	0.208	0.900	0.172	0.156
** r**	-0.058	-0.118	0.139	-0.160	-0.242	-0.054	0.194	0.245	0.027	-0.261	0.292

Significance was considered at the level of p<0.023, Bold represents statistically significant correlations. Abbreviations: FEV_1_: Forced expiratory Volume in one Second, FVC: Forced Exhaled Vital Capacity, DLCO: Diffusing Capacity for Carbon Monoxide, TLC: Total Lung Capacity, FRC: Functional Residual Capacity, RV: Residual Volume, 6MWD: 6 Minute Walking Distance, HRCT: High Resolution Computed Tomography, CPI: Composite Physiological Index, PO_2_: Partial Pressure of Oxygen, FiO_2_: Fraction of inhaled oxygen, SP: Surfactant Protein, IPF: Idiopathic Pulmonary Fibrosis, CPFE: Combined Pulmonary Fibrosis and Emphysema

### SP levels as predictors of survival

**Fig 4:**
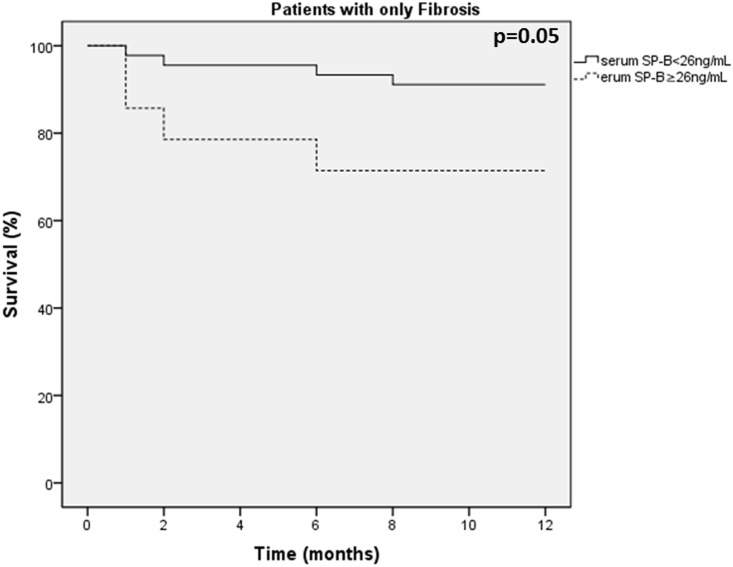
Kaplan-Meier curve showing survival of patients with IPF according to the levels of SP-B.

There were limited deaths during the 1-year follow-up in this study, including 8 patients with IPF and 1 patient with emphysema, whereas no control subjects and no patients with CPFE died during the 1-year follow up. Therefore, the potential predictive role of SP levels on survival was evaluated only in patients with IPF. We have evaluated the role of SP levels as predictors of survival in our patients using the 75^th^ percentiles levels as cut-off points, specifically SP-A ≥280ng/mL, SP-B ≥26ng/mL, SP-C ≥1.9 ng/mL, and SP-D ≥287ng/mL. In patients with IPF, SP-B levels above the 75^th^ percentile of 26 ng/mL were associated with increased mortality (p = 0.05 Log rank test; Fig 4). The levels of SP-A, C and D were not related to survival in patients with IPF (p = 0.642, p = 0.632 and p = 0.623 for SP-A, SP-C and SP-D respectively).

### The effect of smoking on SP levels

**Fig 5:**
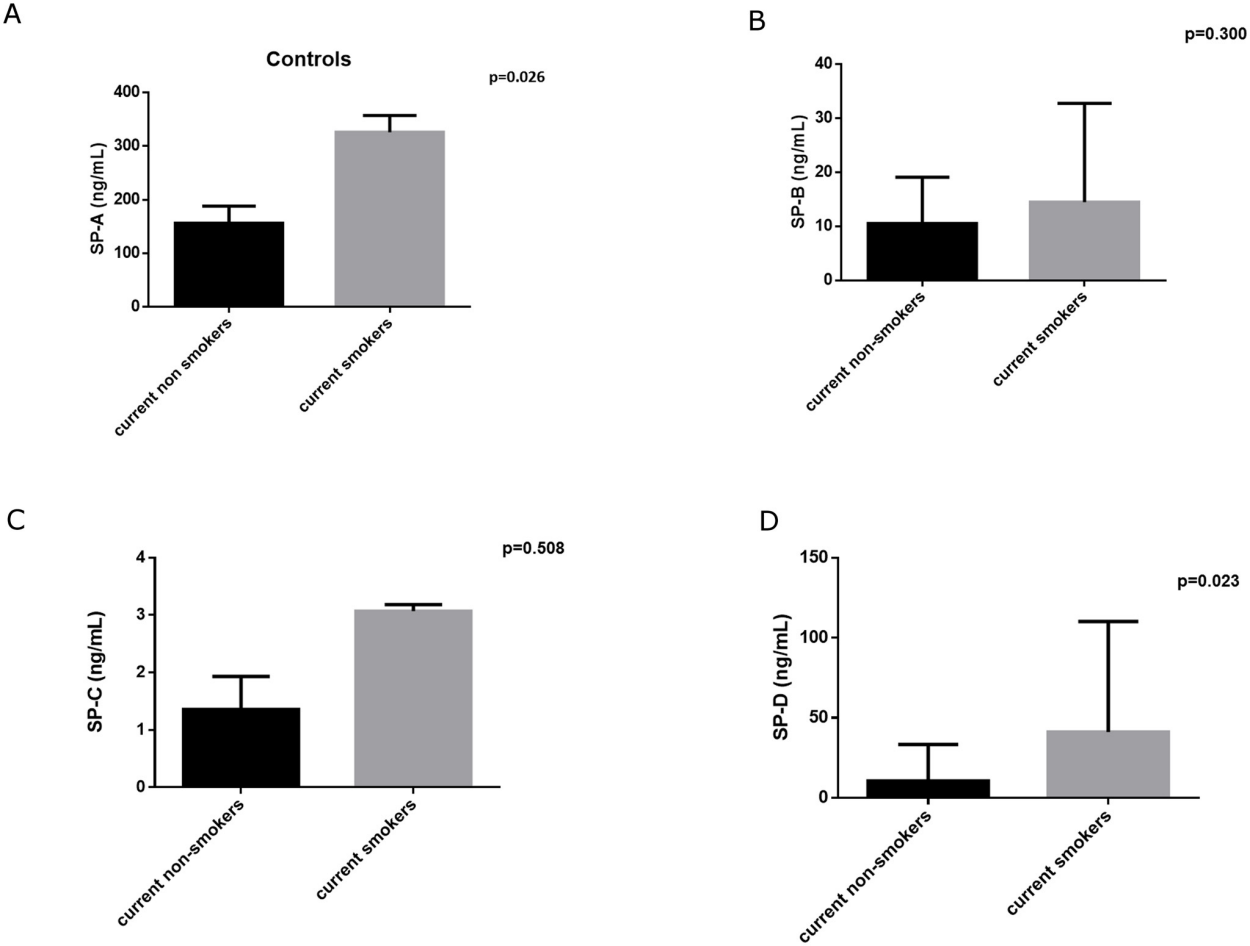
Serum surfactant protein levels in control subjects according to current smoking habit (current non-smokers N = 16 and current smokers N = 9). A. SP-A, B. SP-B, C. SP-C, D. SP-D.

Smoking seemed to influence serum SP-A and SP-D levels only in control subjects. Current smokers had higher serum SP-A and SP-D levels compared to current non-smokers (both never smokers and ex-smokers) [325.7 (152.6, 357.0) vs 156.3 (104.0, 188.2), p = 0.026] and [41.0 (14.3, 110.2) vs 10.4 (5.6, 33.3), p = 0.023 for SP-A and SP-D, respectively]. No differences were observed according to smoking habit regarding SP-B (p = 0.300) and SP-C (p = 0.508) levels (Fig 5A–5D). Furthermore, no significant differences of serum levels of any of the four SPs have been found according to current smoking habit in patients with emphysema, fibrosis or CPFE (data not shown).

### The effect of the extend of fibrosis on SP levels

**Fig 6:**
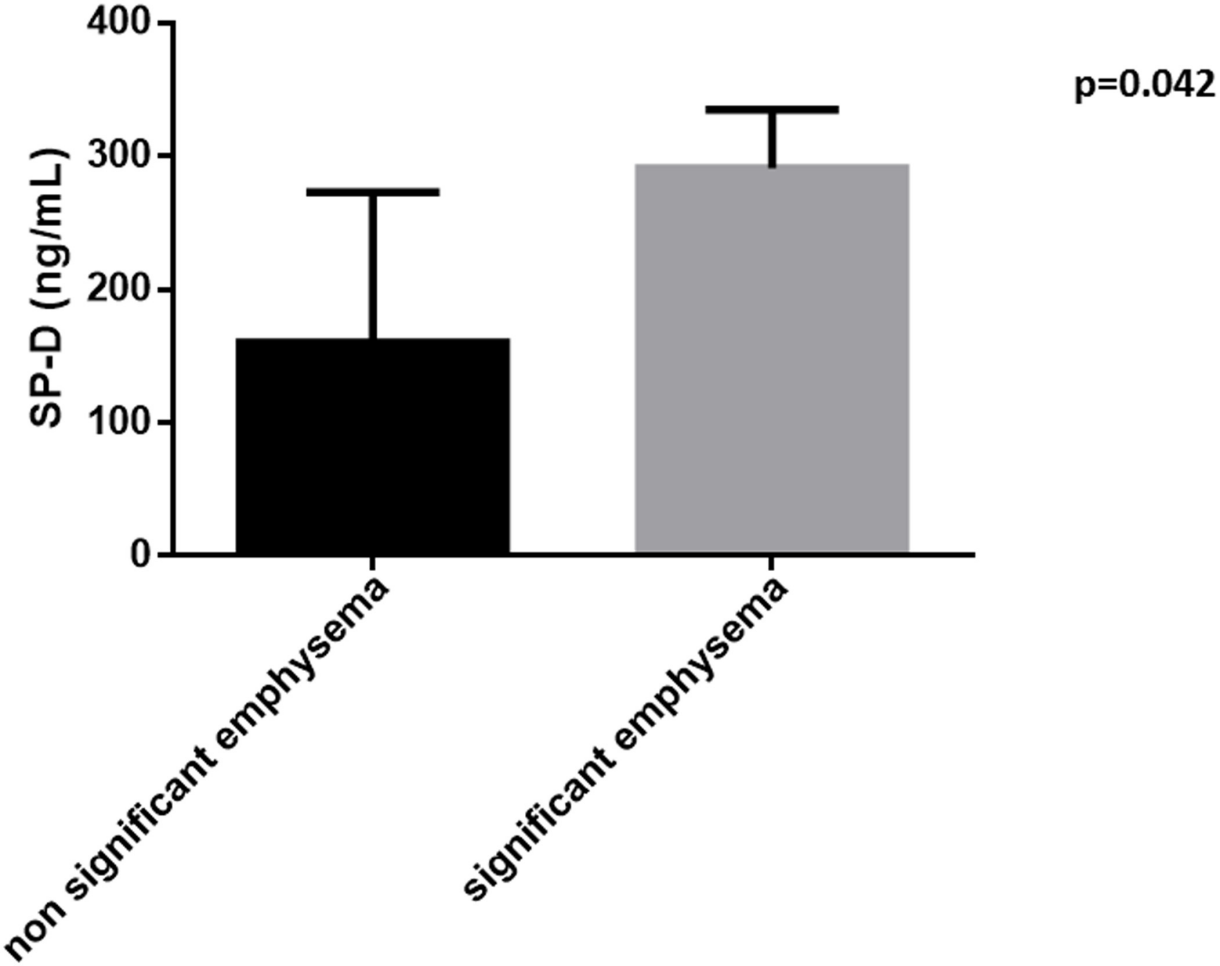
Serum Surfactant Protein levels in CPFE patients according to the extent of emphysematous lesions.

CPFE patients were divided in two subgroups according to the presence of significant extent of emphysematous lesions: 23 patients were considered to have significant emphysematous lesions (i.e. ≥15% of total lung area) whereas 6 patients had emphysematous lesions <15%. SP-D levels were significantly higher in CPFE patients with significant extent of emphysema compared to those without significant extent of emphysema [290.7 (195.5, 334.7) vs 191.5 (37.65, 244.5), p = 0.042] for CPFE patients with and without significant extent of emphysematous lesions respectively (Fig 6). SP-A, SP-B and SP-D levels did not differ between these two groups (p = 0.569, p = 0.309, and p = 0.969 respectively).

## Discussion

In this study we have shown that the levels of SP-A and SP-D differ between controls, patients with emphysema, IPF and CPFE, with IPF and CPFE demonstrating the highest values. Serum SP-D levels were also significantly higher in CPFE patients with significant emphysematous component. In patients with IPF serum SP-A, levels significantly correlated also to CPI a composite index of estimation of disease severity and extent. Furthermore, serum SP-A and SP-D levels were increased in healthy smokers compared to non-smokers. Finally, although SP-B did not differ between groups its blood levels in patients with IPF correlated to FEV_1,_ FVC, DLCO, FRC and TLC and patients in the upper quartile of SP-B values were associated with worse survival. To our knowledge, this is the first study examining the possible differences of the levels of the four SPs in these four groups of patients.

Regarding SP-A and SP-D, our findings confirm previous studies that have shown increased levels in the circulation [24] of patients with fibrosis, although these levels were decreased in the BAL fluid [22, 47] but without a significant association between the circulating levels of these proteins and the extent of fibrosis in radiographic studies, a finding that was also confirmed in our study. Our finding that serum SP-A and SP-D levels are higher in patients with fibrosis (IPF or CPFE) compared to those with only emphysema, suggests that the combination of alveolar parenchymal derangement that characterizes fibrosis with the hyperplasia of type II alveolar cells [48], may lead to the translocation of greater amounts of these SPs from the lung parenchyma to the peripheral circulation through the pulmonary vasculature in these patients.

However, the fact that patients with CPFE and more extensive emphysematous lesions in HRCT also expressed significantly higher levels of both SP-A and SP-D, might suggest that the emphysematous component on top of the fibrotic process, stretched and overdistended by the nearby fibrotic lesions, also contributes through an alveolar damage to the elevation of SP levels in these patients [49–51]. Interestingly, in our study circulating levels of SP-A, correlated to CPI in patients with IPF, CPI being a composite score found to be an important indicator of the total respiratory impairment in IPF and related to the total extent of fibrosis in these patients [37]. Although in patients with fibrosis, CPI has been acknowledged as a predictor of survival, this was not the case in patients with CPFE [36], which probably explains the lack of any significant correlation of SPs with CPI in this group in our study.

Furthermore, in our control group, we have observed that healthy current smokers had higher serum levels of SP-A and SP-D compared to current non-smokers (including both never and ex-smokers). This is in accordance to previous studies showing that smokers had decreased levels of SP-A and SP-D in the bronchoalveolar lavage (BAL) fluid [52] although they seemed to express higher circulating levels of the former [53]. A possible explanation of these findings was that smokers may present an alveolo-capillary leakage of surfactant proteins into the blood [54]. The fact that serum levels of SP-A are higher in current smokers compared to ex-smokers [55], also observed in our cohort, might also represent the ongoing lung inflammation caused by cigarette smoke which leads to increased vascular permeability and to SP-A and SP-D translocation from the pulmonary compartment to the circulation [56]. It is known that smoking cessation seems to rapidly restore the alveolar-capillary barrier integrity [57] and this is probably the reason why healthy ex-smokers presented with similar serum levels of SP-A and SP-D. However, this was not the case in patients with emphysema, fibrosis or CPFE in which circulating SP levels did not differ between current smokers and current non-smokers and it seems that the established lung inflammation in both pulmonary emphysema [58–61] and fibrosis [62–65] continues despite smoking cessation. Increased serum SP-A and SP-D levels in current smokers might be an early indicator of an ongoing parenchymal damage which might lead to the development of lung disease.

Regarding SP-B, although its blood levels did not differ among groups, in patients with fibrosis we found a correlation with both individual indices of disease severity and extent such as FVC and DLCO, and in addition a marginal difference in survival, with patients with SP-B levels at the higher quartile presenting lower survival. This finding is in accordance to previous studies showing the relation of SP-B with the development of interstitial lung disease in both animals [66] and humans [67]. FVC and DLCO are known to be significant predictors of survival in IPF [68, 69] and this might explain the weak association between serum SP-B levels and mortality in this group of patients in our study. The mortality findings related to the increased SP-B levels, however, need to be further validated in larger studies.

Finally regarding SP-C there is some evidence that might play a role in the development of CPFE [30, 31] and patients with lung disease comparable with CPFE were found to have mutations in the SP-C gene [32, 33]. It is important to mention that in our group of patients, serum levels of SP-C did not differ in patients with CPFE and were not associated with any disease characteristic including pulmonary function impairment, and the extent of fibrosis. The lack of such an association might relate to fact that SP-C gene mutations possibly result to functional rather than quantitative alterations of SP-C [70].

Our study has significant limitations. First, we have used an observational method for the quantification of fibrosis and/or emphysema in HRCT, instead of dedicated CT software. However, this method presents excellent correlation with densitometry quantitation [39, 40] and can be performed in everyday clinical practice. Second, patients were followed up for only one year which is probably the reason for the lack for any significant associations regarding SPs and survival in patients with emphysema and CPFE. Finally, it is a fact that SP-A and SP-D are additionally produced in several extrapulmonary locations, including the brain, the salivary glands, the heart, the kidneys and the reproductive tract [71] and for this reason we cannot confirm that the levels that are measured in serum directly originate from the lung. However, since the greater amounts of all four surfactant proteins are synthesized by alveolar type II cells and SP-A, SP-B and SP-D are also produced by different types of airway cells, including Clara cells and submucosal cells, we can conclude that the major source of serum SP levels comes from the pulmonary epithelium.

In conclusion, in this study including controls, emphysema, IPF, and CPFE patients we have shown that serum SP- A and SP-D present significantly higher serum levels where fibrosis exists or coexists while serum SP-A levels are also related to fibrosis severity and extent as reflected by the CPI score of estimation of disease severity. The above findings might suggest that serum SP-A and SP-D levels are related to the type of alveolar damage installed on fibrotic lungs and reflect their overproduction due to hyperplasia of type II alveolar cells, or overleakage in the systemic circulation caused by alveolar basement membrane splintering off and hyperpermeability. In IPF also SP-B relates to survival. Further studies are needed to define the role of the above SPs as markers of disease severity.
